# Protein oxidation mediated by heme-induced active site conversion specific for heme-regulated transcription factor, iron response regulator

**DOI:** 10.1038/srep18703

**Published:** 2016-01-05

**Authors:** Chihiro Kitatsuji, Kozue Izumi, Shusuke Nambu, Masaki Kurogochi, Takeshi Uchida, Shin-Ichiro Nishimura, Kazuhiro Iwai, Mark R. O’Brian, Masao Ikeda-Saito, Koichiro Ishimori

**Affiliations:** 1 Department of Chemistry, Faculty of Science, Hokkaido University, Sapporo 060-0810, Japan; 2 Department of Molecular Engineering, Graduate School of Engineering, Kyoto University, Kyoto 615-8510, Japan; 3 Institute of Multidisciplinary Research for Advance Materials, Tohoku University, Sendai 980-8577, Japan; 4 Faculty of Advanced Life Science, and Graduate School of Life Science, Hokkaido University, Sapporo 001-0021, Japan; 5 Graduate School of Frontier Biosciences, Osaka University, Suita 565-0871, Japan; 6 CREST, Japan Science and Technology Agency, Tokyo 103-0027, Japan; 7 Department of Microbiology and Immunology, State University of New York at Buffalo, Buffalo, New York 14214, United States.

## Abstract

The *Bradyrhizobium japonicum* transcriptional regulator Irr (iron response regulator) is a key regulator of the iron homeostasis, which is degraded in response to heme binding via a mechanism that involves oxidative modification of the protein. Here, we show that heme-bound Irr activates O_2_ to form highly reactive oxygen species (ROS) with the “active site conversion” from heme iron to non-heme iron to degrade itself. In the presence of heme and reductant, the ROS scavenging experiments show that Irr generates H_2_O_2_ from O_2_ as found for other hemoproteins, but H_2_O_2_ is less effective in oxidizing the peptide, and further activation of H_2_O_2_ is suggested. Interestingly, we find a time-dependent decrease of the intensity of the Soret band and appearance of the characteristic EPR signal at g = 4.3 during the oxidation, showing the heme degradation and the successive formation of a non-heme iron site. Together with the mutational studies, we here propose a novel “two-step self-oxidative modification” mechanism, during which O_2_ is activated to form H_2_O_2_ at the heme regulatory motif (HRM) site and the generated H_2_O_2_ is further converted into more reactive species such as ·OH at the non-heme iron site in the His-cluster region formed by the active site conversion.

Iron is an essential nutrient for almost all organisms, playing a central role in a large number of crucial cellular functions, such as oxygen transport, chemical catalyst for xenobiotic metabolism, mitochondrial energy generation, and electron transport^1^. However, excessive intracellular iron can be toxic because of its ability to convert molecular oxygen into reactive oxygen species (ROS) that damage cellular components. Thus, iron homeostasis is strictly regulated so that iron acquisition, storage, and consumption are balanced by iron availability. In addition, regulated homeostasis prevents intracellular levels of free iron from reaching toxic levels^2^. Defects in iron homeostasis results in severe diseases such as anemia, pica, liver cirrhosis and neurological deficits.

Despite the biological significance, the regulation mechanism based on the molecular structures of the key proteins in iron homeostasis remains unknown and the functional characterizations of these proteins are also still quite limited. To clarify the molecular mechanisms, we have focused on nitrogen-fixing bacteria, which live as endosymbionts in root nodules of plants. In this type of bacteria, a high expression of iron- or heme-containing proteins is required to promote nitrogen fixation. The nitrogenase complex, which comprises more than 10% of the total cellular protein of nitrogen-fixing bacteroids, contains 30–34 iron atoms per molecule^3^. A previous study demonstrated that a nitrogen-fixing bacterium, *Bradyrhizobium japonicum*, expresses a sensor protein, iron response regulator (Irr), to regulate iron homeostasis and metabolism^4^.

Irr is a key regulator of iron homeostasis that functions under low iron conditions to regulate gene expression^4^. When the cells are grown in limiting iron concentrations, Irr acts as a repressor for the transcription of the *hemB* gene encoding one of the heme biosynthetic enzymes, δ-aminolevulinic acid dehydratase, to prevent accumulation of a heme precursor, protoporphyrin IX. In response to cellular exposure to iron, Irr degrades, allowing *hemB* transcription to resume and promoting heme biosynthesis^5^. Irr forms a complex with the heme biosynthetic enzyme ferrochelatase, and therefore, Irr responds to the status of heme at the site of synthesis^6^. Our previous studies show that Irr has two heme binding sites^5^,^7^,^8^, and this binding is supposed to trigger Irr degradation by a mechanism that involves oxidative modification of the protein.

Although the oxidation of Irr is thought to be promoted by the activation of molecular oxygen by heme molecule bound to Irr^9^, and the degradation of Irr would involve the redox activity of heme and oxidation of the protein^9^,^10^, the molecular mechanism of this heme-mediated self-oxidation is yet to be elucidated. The ROS responsible for the oxidative modification of Irr, as well as the sites of this modification, also remain unidentified.

To this end, we focused on the active species for the oxidative modification of Irr and examined the oxidation reaction in the presence of ROS scavengers by the Oxyblot analysis^11^. We also utilized EPR and mass spectroscopies to identify the active center for the ROS generation and oxidation sites in Irr. Based on these experimental results, we discussed the heme-mediated ROS formation and oxidation mechanism in Irr and proposed the “active center conversion” from heme iron to non-heme iron in the protein to facilitate a novel two step oxygen activation from molecular oxygen to highly reactive oxygen species.

## Results

### Oxidation Reaction of Irr and Active Species for the Oxidation

**Figure 1:**
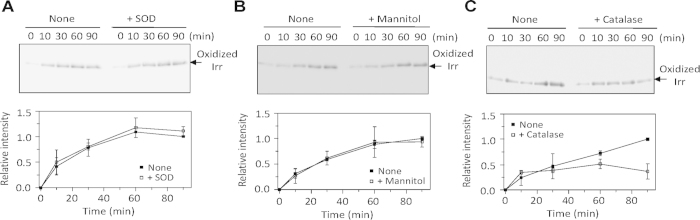
Heme-mediated oxidation of Irr. The time-course Oxyblot assay of the oxidation of Irr (20 μM) by the aerobic incubation with DTT (2 mM) and heme in the presence or absence of ROS scavengers; (**A**) 500 units/ml SOD, (**B**) 20 mM mannitol or (**C**) 1,000 units/ml catalase. A western blot for oxidized Irr (upper panel) and a time course of the normalized intensity (lower panel). The data points and error bars represent the means and standard deviation of three independent experiments using three different protein preparations.

In the oxidative modification of proteins, the identification of the active oxygen species and specific oxidation sites is essential to understand the molecular mechanism. To identify the active species for the oxidative modification of Irr, we examined the oxidation reaction of Irr using Oxyblot^11^ in the presence of specific ROS scavengers^12^. In the Oxyblot analysis, the oxidized proteins, typically carbonylated proteins, can be detected by Western blotting^10^. In the samples with no ROS scavengers (upper panels of Fig. 1A–C), a band appeared upon addition of the reductant in the presence of heme under aerobic conditions, showing that the heme-bound Irr is oxidized in the presence of molecular oxygen and the reductant, DTT. The band intensity of oxidized Irr increased over time, confirming the oxidation of Irr proceeded in the presence of heme and DTT.

The addition of the superoxide anion (O_2_^−^) scavenger (superoxide dismutase: SOD) or the hydroxyl radical (·OH) scavenger (mannitol) did not affect the time-dependent intensity of the band for oxidized Irr (upper panels in Fig. 1A,B) and did not suppress the oxidation of Irr (lower panels in Fig. 1A,B). This implies that O_2_^−^ and ·OH are not generated during the oxidation of Irr or that these scavengers fail to inhibit the oxidation of the amino acid residues due to the close location of the target amino acid residues to the ROS generation sites^13^. However, the H_2_O_2_ scavenger, catalase, significantly repressed the oxidation of Irr (Fig. 1C), indicating that H_2_O_2_ was generated in the oxidation reaction of Irr.

### Spectroscopic Detection of H_2_O_2_ in the Oxidation Reaction of Irr

**Figure 2:**
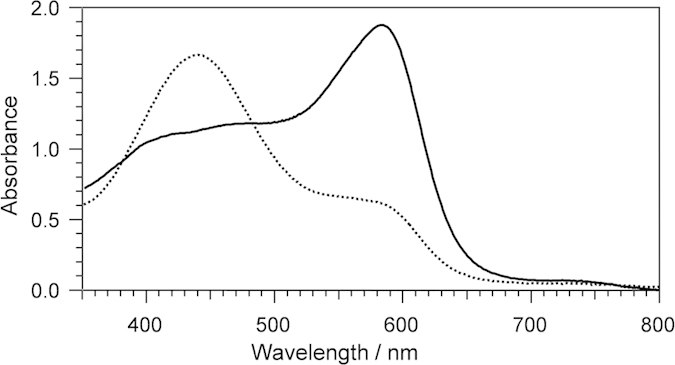
Spectroscopic assay for H_2_O_2_ generation by Irr. Absorption spectral changes for the H_2_O_2_ generation assay in the presence (solid line) and absence of Irr (dotted line). The reaction solution contains 125 μM xylenol orange and 25 mM FeCl_2_. After 15 min incubation at room temperature, the absorption spectra were measured and the amount of generated H_2_O_2_ was quantified by the absorbance at 595 nm.

We further confirmed the generation of H_2_O_2_ by a spectroscopic assay employing xylenol orange, which is based on the Fenton reaction^14^. In this assay, H_2_O_2_ oxidizes Fe^2+^ to form the Fe^3+^ -xylenol orange complex, which can be quantified by an absorbance at 595 nm^14^. As shown in Fig. 2, the absorbance at 595 nm was increased with the addition of heme-bound WT Irr in the presence of DTT, confirming the formation of H_2_O_2_ (Table 1). The addition of catalase drastically suppressed the formation of H_2_O_2_ (Table 1), which further confirmed the formation of H_2_O_2_ in the oxidation of Irr.

**Figure 3:**
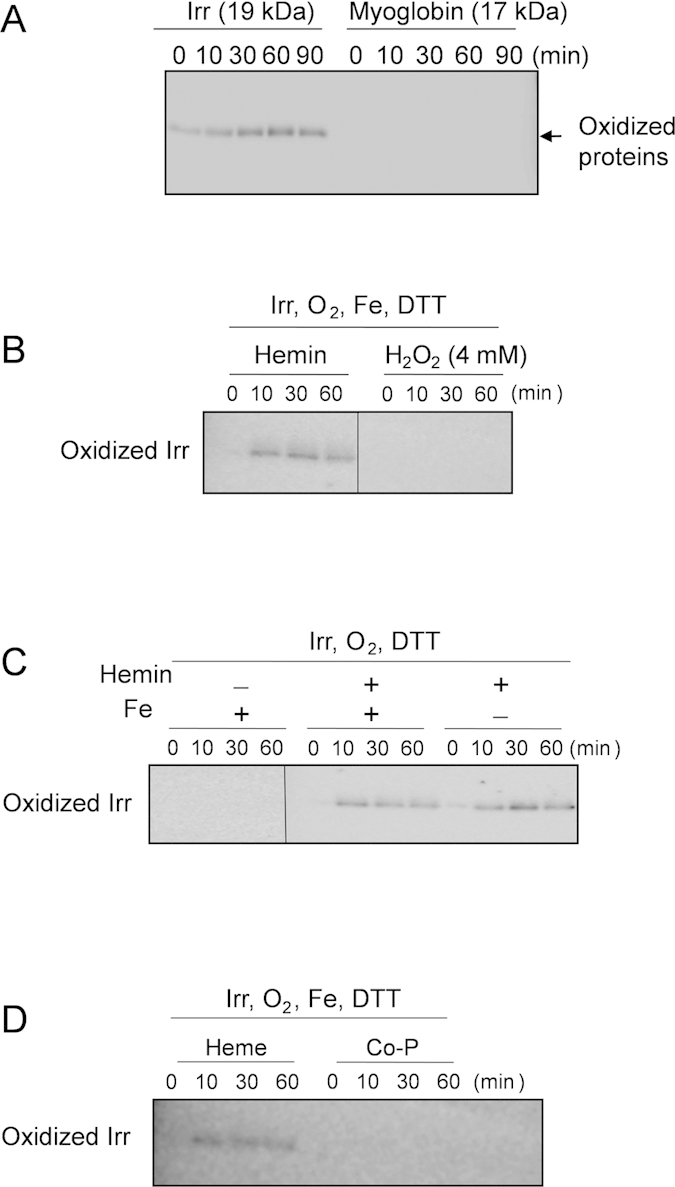
Oxyblot assay for heme-mediated oxidation reaction in Irr and myoglobin. The time-course Oxyblot of the oxidation of 20 μM Irr or myoglobin under the aerobic condition with 2 mM DTT. (**A**) The oxidation reactions of Irr or myoglobin. (**B**) The oxidation reactions of Irr in the presence of heme or 4 mM H_2_O_2_ and (**C**) in the presence or absence of exogenous 200 μM ferrous iron with heme. (**D**) The oxidation reactions of Irr in the presence of two equivalent of heme and cobalt porphyrin.

In the absence of either heme or reductant (DTT), the generation of H_2_O_2_ was not detected (Table 1), demonstrating that the oxidation of Irr requires both heme and reductant as previously suggested^9^. Because the formation of H_2_O_2_ from molecular oxygen in the presence of a reductant is one of the common reactions of hemoproteins^15^, we were also able to confirm the generation of H_2_O_2_ in the reaction of myoglobin, a typical hemoprotein, or BSA, a typical heme-binding protein, with molecular oxygen in the presence of a reductant (Table 1). However, myoglobin was not carbonylated under the same conditions in which Irr was oxidized (Fig. 3A). In addition to these His-ligated heme-bound proteins, Cys-ligated proteins such P450 also reacts with H_2_O_2_ to form high valent iron species, but the oxidative modifications of the proteins were not reported^16^,^17^,^18^. Thus, the oxidative modification is specific for Irr, not for conventional hemoproteins such as myoglobin and P450.

### Identification of the H_2_O_2_ Generation Site

As we previously reported, Irr had two heme binding sites^9^, which were the potential H_2_O_2_ generation sites. One of the heme binding sites is the HRM (-^29^Cys-^30^Pro-) in the N-terminal region of Irr, and the other is located at the His-cluster region (-^117^His-^118^His-^119^His-)^5^,^9^. The mutation of ^29^Cys to Ala resulted in the loss of one of the heme binding sites^7^ and the degradation rate of the ^29^Cys mutant Irr was severely reduced^5^. On the other hand, the protein degradation of a mutant with Ala residues at the positions of ^117^His, ^118^His and ^119^His in the His-cluster region was also completely suppressed^9^. Based on these observations, we previously concluded that the iron-dependent degradation of Irr involved two heme binding sites^9^.

To determine the H_2_O_2_ generation site in Irr, we measured the H_2_O_2_ generation by the absorption change at 595 nm for the two mutants: the HRM (C29A: ^29^Cys → Ala) and His-cluster (H117-119A: ^117^His → Ala, ^118^His → Ala, ^119^His → Ala) mutants in the presence of xylenol orange and ferrous iron (Table 1). While the His-cluster mutant was still able to generate H_2_O_2_ (31.3 μM) at comparable level to the wild type protein (33.4 μM), the generation of H_2_O_2_ was decreased in the HRM mutant (C29A, 16.5 μM) (Table 1). The suppressed H_2_O_2_ production in the HRM mutant corresponds to the slow heme-dependent protein degradation that was previously reported for the C29A mutant^9^. The lower amount of the generated H_2_O_2_ in the HRM mutant than that in the His-cluster mutant implies that the primary H_2_O_2_ generation site is the heme bound at the HRM. As we reported previously^7^, heme in the His-cluster region is in the six-coordinate state, while heme in the HRM region is in the five-coordinate state. The ligation of the sixth ligand (His) to the heme iron in the His-cluster region would reduce the affinity of H_2_O_2_ to the heme iron, resulting in the lower generation of H_2_O_2_ in the heme binding site of the His-cluster region.

### Further Activation of H_2_O_2_ in the Oxidation of Irr

While the heme bound in the HRM region is shown to be the primary active center for the generation of H_2_O_2_ in the oxidation reaction of Irr, H_2_O_2_ is a rather moderate ROS that cannot directly oxidize amino acid residues^19^. Even though myoglobin, a typical hemoprotein, can more efficiently convert molecular oxygen into H_2_O_2_ than Irr (Table 1)^15^, it was not carbonylated at all under the aerobic conditions (Fig. 3A). Although the reaction of heme with H_2_O_2_ results in the formation of the high valent reaction intermediates such as ferryl-oxo porphyrin and/or porphyrin radicals, these intermediates are still not so highly reactive species that can directly oxidize amino acid residues as found for Irr^19^. The preferential oxidative modification of Irr strongly suggests that Irr has a specific reaction mechanism that can activate H_2_O_2_ to generate more reactive species, such as ·OH, for the oxidation of Irr.

One of the activation reactions for H_2_O_2_ is the Fenton type reaction (H_2_O_2_+Fe^2+ →^·OH + OH^−^ + Fe^3+^)^20^. In the presence of ferrous iron, H_2_O_2_ is activated to form ·OH, which could readily oxidize amino acid residues to form the carbonyl adducts. However, no significant amounts of iron and other transition metal ions were detected by the inductively coupled plasma atomic emission spectrometry (ICP-AES) measurements of Irr (Table S1 in Supplementary Information). As depicted in Fig. 3B, the simple addition of H_2_O_2_ did not induce the oxidation of Irr in the absence of heme, suggesting that heme-free Irr cannot activate H_2_O_2_ by the Fenton type reaction and supporting no iron binding in Irr before the addition of heme. The absence of oxidative modification in the presence of exogenous iron and reductant in the absence of heme (left image of Fig. 3C) suggests that the exogenous iron cannot bind to Irr to form an H_2_O_2_ activation site. The addition of exogenous iron did not enhance the oxidation of Irr in the presence of heme (middle and right images of Fig. 3C), confirming that the exogenous iron ion cannot participate in the oxidative modification of Irr.

### Heme Degradation and Active Site Conversion to Non-heme Iron Binding Site

As previously reported^19^, the addition of H_2_O_2_ to hemoproteins leads to the heme degradation and release of iron from the heme molecule, and this H_2_O_2_-mediated heme degradation and iron release was enhanced in some proteins^21^. The released iron can react with H_2_O_2_ via the Fenton type reaction to generate ·OH, a highly reactive oxygen species that could promote the oxidative modification of amino acids. Considering that heme is bound in a His-cluster region and His is a typical ligand for non-heme iron, it is likely that the iron released from the degraded heme molecule is readily trapped by histidines of Irr, resulting in the active site conversion to a non-heme iron binding site in the protein matrix and activating H_2_O_2_ to generate ·OH. Addition of 1 mM EDTA failed to inhibit the oxidative modification of Irr, also showing that iron released from heme was captured by amino acid residues interior of the protein without diffusing to the solvent.

**Figure 4:**
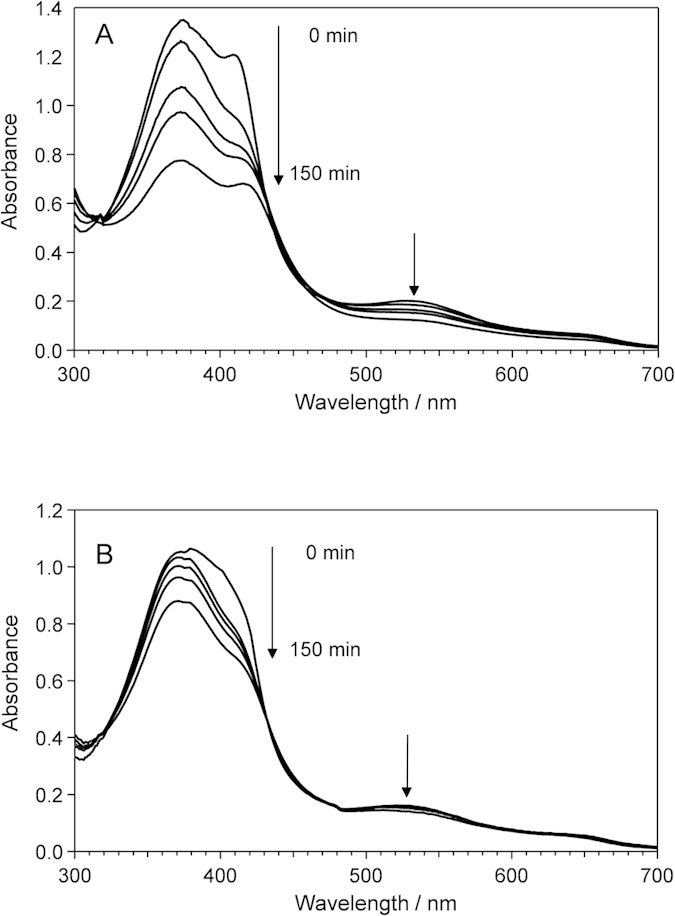
Absorption spectral changes in the oxidation of Irr. The oxidation reaction was initiated by addition of 2.0 and 1.0 equivalent heme to 500 μL of (**A**) 20 μM Irr and (**B**) 15 μM His-cluster (H117-119A) mutant, respectively, in the presence of 2 mM DTT. After 0, 10, 30, 60 and 150 min incubation, the UV-visible absorption spectra of the sample were measured.

**Figure 5:**
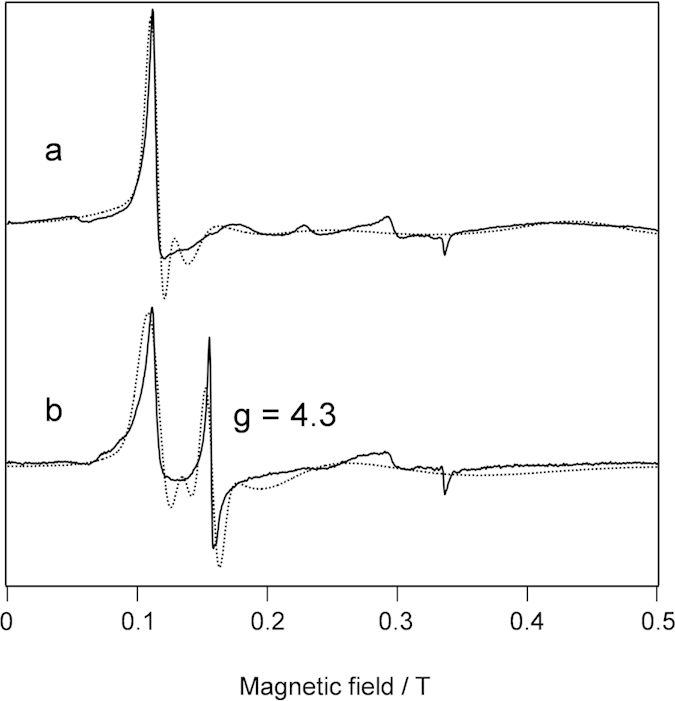
EPR spectra of Irr in oxidation reaction. The oxidation reaction was initiated by addition of 1.0 equivalent heme to 500 μL of the Irr solution (1 mM Irr, 2 mM DTT). (**a**) immediately after the addition of heme, and (**b**) after 6 hours incubation at room temperature. The observed and simulated spectra are shown in the solid and dotted line, respectively. The details of the simulation is described in Supplementary Information.

To corroborate the heme degradation in the oxidation of Irr, we followed the absorption spectral changes of heme-bound Irr in the presence of the reductant. As evident in Fig. 4A, during the oxidation of Irr, a decrease of the absorbance in the Soret region was observed, implying that the heme molecule undergoes degradation. Concomitant with the degradation of heme, heme-bound Irr in the oxidation reaction showed an EPR signal at *g* = 4.3, which is characteristic of non-heme iron (Fig. 5b), confirming the release of iron from heme and formation of a non-heme iron site.

To assess the stoichiometry of the conversion of the heme iron to the non-heme iron in heme bound Irr, the EPR spectra were integrated to estimate the heme iron and non-heme iron contents before and after the oxidation. The integrated EPR spectra (Figure S1 in Supplementary Information) were fitted by the 5-component Gauss functions and parameters for the best fittings were determined by the non-linear least square method. The relative concentrations in the Irr samples were estimated by the coefficient for the Gaussian functions (Table S3 in Supplementary Information). By the oxidation, the relative content of the high spin heme was decreased from about 60% to 40%, and that of the low spin heme was also decreased from about 30% to 15%. Assuming that the iron released from the heme degradation forms the non-heme iron binding site, the decrease in the relative contents of the high and low spin hemes suggests that the relative content of the non-heme iron would be about 35%. On the other hand, after the oxidation, the quantification of the EPR signals showed that the relative contents of the non-heme iron is about 40%. Considering the contribution of the unknown species to the relative contents, the relative content of non-heme iron estimated from the decrease in the relative contents of the high and low spin heme species by the oxidation is almost identical to the observed increase in the relative contents of non-heme iron after the oxidation. This estimation confirms that iron for the non-heme iron binding site formed in the oxidation reaction are derived from iron released from the heme degradation in heme bound Irr.

### Identification of the Oxidation Site

**Figure 6:**
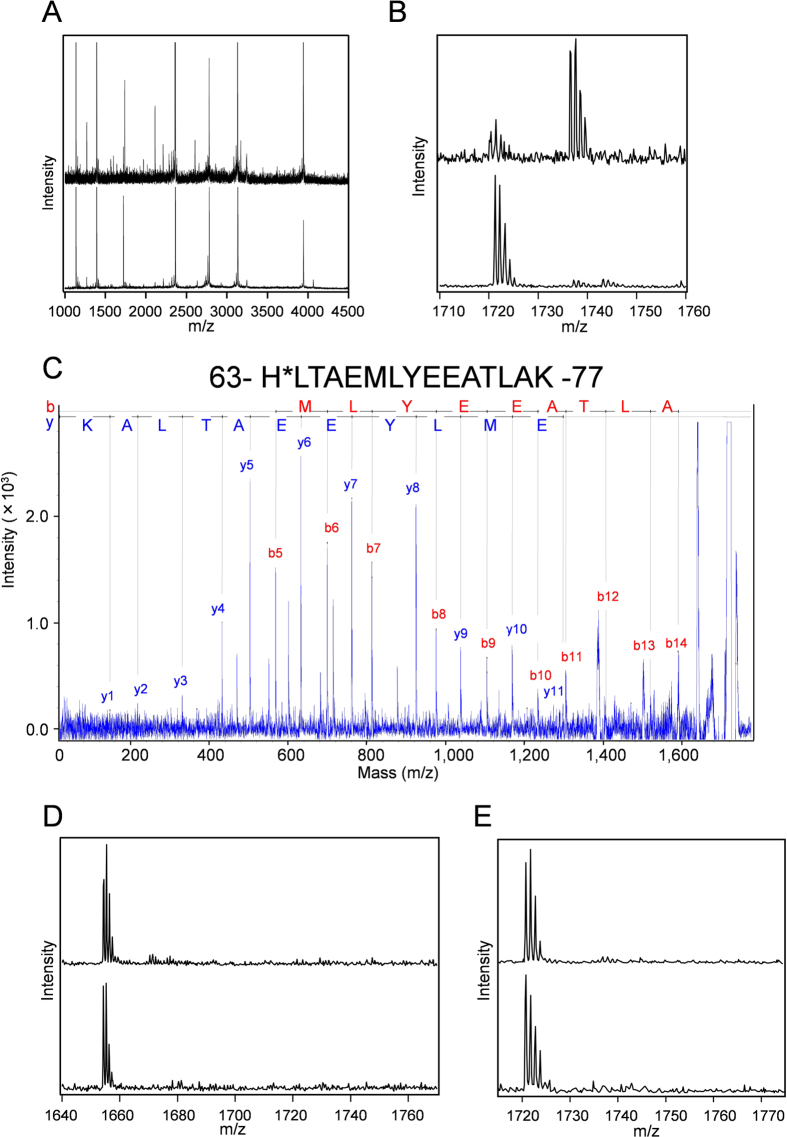
MALDI-TOF and MALDI-TOF/TOF spectra of the digested Irr. MALDI-TOF mass spectra of the tryptic-digested peptide fragments of Irr (40 μM) (**A**) 1000–4500 m/z, (**B**) 1710–1760 m/z before oxidation (lower spectrum) or after oxidation (upper spectrum). (**C**) Carboxyl-terminal (y-series) and amino-terminal (b-series) fragmentation products are shown. In the Irr 63–77 sequence, the asterisk shows the oxidation of a residue of Irr (^63^His). MALDI-TOF mass spectra of the tryptic-digested peptide fragments of the His cluster mutants (40 μM) (**D**) Irr (H117-119A), (**E**) Irr(H63A) before oxidation (lower spectrum) or after oxidation (upper spectrum).

To identify the specific site of oxidation in the heme-bound Irr, we carried out MALDI-TOF mass spectrometry on the tryptic-digested fragments of Irr before and after the oxidation reaction. By a comparison of these mass spectra, we successfully detected a new mass peak associated with the oxidation of Irr (Fig. 6A). In the mass spectrum between *m/z* 1710 to *m/z* 1760 (Fig. 6B), a new mass peak at *m/z* 1736 appeared (upper spectrum in Fig. 6B), corresponding to the oxidized peptide fragment with the incorporation of one oxygen atom to the mass peak at *m/z* 1720 (lower spectrum in Fig. 6B). Based on the amino acid sequence of Irr, the mass peak from *m/z* 1720 can be assigned to the peptide fragment consisting of amino acids ^63^His-^77^Lys (calculated mass is 1719.9 Da). The appearance of the oxidized peak indicates that one of the amino acid residues in the ^63^His-^77^Lys peptide fragment was oxidized in the oxidation reaction.

To determine the specific residue that is oxidized in the ^63^His-^77^Lys fragment, we analyzed the TOF/TOF mass spectrum of the peak at *m/z* 1736. As shown in Fig. 6C, we found that the residues between ^67^Glu and ^77^Lys were intact and that one of the amino acid residues between ^63^His and ^66^Ala was oxidized. Among these residues (^63^His-^64^Leu-^65^Thr-^66^Ala), histidine is the most labile residue to oxidation reactions^22^, and the metal-catalyzed oxidation of histidine residues to form 2-oxohistidine has also been reported for Cu, Zn-SOD in the presence of H_2_O_2_^23^, leading to our conclusion of ^63^His as one of the oxidation sites of Irr. The oxidative modification at His63 was supported by the mass spectrum of the Irr(^63^His → Ala)(H63A) mutant. As illustrated in Fig. 6D, the mass spectrum of the H63A mutant indicates that the ^63^Ala-^77^Lys fragment was not oxidized and ^63^His is the oxidative modification site.

### Identification of the H_2_O_2_ Activation Site in Irr

**Figure 7:**
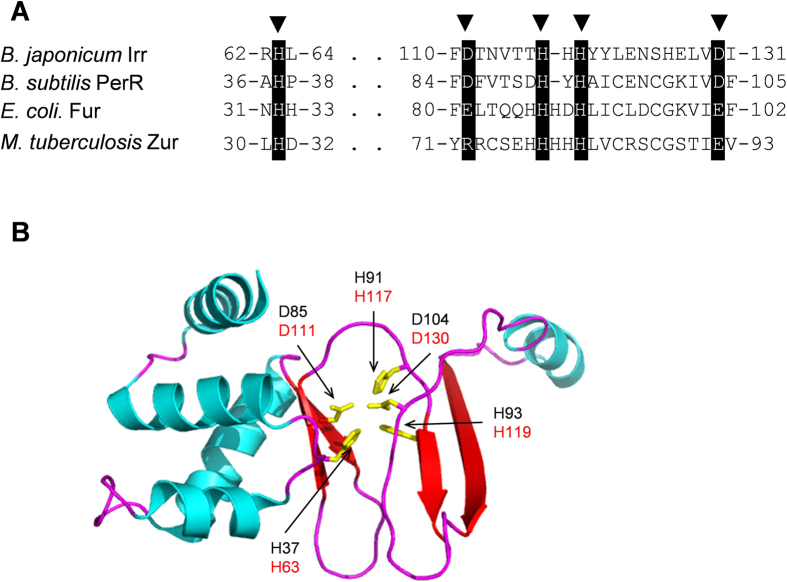
The sequence alignment of *Bradyrhizobium japonicum* Irr and *Escherichia coli* Fur family proteins^24^ and the X-ray structure of monomer *Bacillus subtilis* PerR (Protein Data Bank code 3F8N)^28^. (**A**) In the sequence alignment, a black background marks sequence identity, and the wedges show the predicted iron binding sites. (**B**) In the protein structure, ligands for non-heme iron are shown in yellow. The black and red residue numbers are correspond to PerR and Irr, respectively.

The possible formation of 2-oxohistidine by the reaction of ROS with the His residues around the non-heme iron center suggests that ^63^His is one of the ligands for the non-heme iron or located adjacent to the non-heme binding site. It should be noted here that most of the Fur family proteins have the “His-cluster” regions consisting three histidines and two glutamic acids. Based on the sequence homology in the Fur family proteins (Fig. 7A)^24^, ^63^His corresponds to one of the histidines constituting the proposed non-heme binding site, and the other two histidines, ^117^His and ^119^His, both of which are located in the His cluster region, are predicted to be the other ligands for the non-heme binding site, showing that non-heme binding site to activate H_2_O_2_ is located in the His-cluster region (Fig. 7B).

To confirm that the His-cluster region is the non-heme binding site that can activate H_2_O_2_ in Irr, we followed the spectral changes in the mass spectrum of the His-cluster mutant (H117-119A: ^117^His → Ala, ^118^His → Ala, ^119^His → Ala) in the oxidation reaction. Figure 6E clearly shows that ^63^His in the His-cluster mutant is intact under the conditions in which wild type Irr is oxidized (upper spectrum in Fig. 6A,B) and the time-dependent changes in the absorption spectrum for the His-cluster mutant in the presence of heme under aerobic conditions was significantly small (Fig. 4B), indicating that the heme degradation was suppressed to inhibit the formation of the non-heme iron binding site in the His-cluster region of Irr.

The formation of the H_2_O_2_ activation site in the His-cluster region by the released iron from heme was further confirmed by the fact that there was no oxidation of Irr when cobalt porphyrin was bound to the protein (Fig. 3D). Therefore, we propose that the iron that is released from heme is ligated by histidines in the His-cluster region to catalyze the activation of H_2_O_2_, which generates highly reactive oxygen species, such as ·OH, leading to the oxidative modification of amino acids in the His cluster region.

## Discussion

The present results showed that the “active site conversion” during the oxidative modification in Irr, where the heme degradation is induced in the presence of the reducing reagent and iron ion released from the heme forms a non-heme binding site in the His cluster region without diffusing to the solvent. The non-heme binding site formed in the His-cluster region would be a H_2_O_2_ activation site to generate more reactive oxygen species such as ·OH. ^63^His, one of the histidine residues supposed to be ligated to the non-heme iron, is oxidized to 2-oxohistidine by the reaction with highly reactive oxygen species activated from H_2_O_2_ at the non-heme binding site.

A similar H_2_O_2_-mediated oxidative modification was also described for the bacterial H_2_O_2_ sensor PerR^25^. PerR is a member of the ferric uptake regulator (Fur) family of proteins including Irr^26^. The addition of H_2_O_2_ induces the oxidative modification of residues within PerR, leading to the degradation of the protein, similar to Irr^27^. In this H_2_O_2_-mediated oxidation mechanism in PerR, H_2_O_2_ reacts with the non-heme iron intrinsically bound in PerR, where the iron is bound to ^37^His, ^85^Asp, ^91^His, ^93^His and ^104^Asp of PerR, and H_2_O_2_ is converted into ·OH through the Fenton reaction^25^. The generated ·OH then oxidizes the iron liganded histidines (^37^His and ^91^His), resulting in the destabilization of the protein structure and loss of the DNA binding activity^25^. The amino acid residues constituting the non-heme iron binding site in *Bacillus subtilis* PerR (^37^His, ^85^Asp, ^91^His, ^93^His and ^104^Asp) are completely conserved in the His-cluster region of *B. japonicum* Irr (^63^His, ^111^Asp, ^117^His, ^119^His and ^130^Asp) (Fig. 7A), which supports the non-heme iron site in the His-cluster region and activation of H_2_O_2_ to highly reactive oxygen species such as ·OH in the His-cluster region of Irr.

In contrast to the close similarity of the H_2_O_2_-mediated oxidation mechanism to PerR, the oxidation in Irr was less specific than that in PerR. Many new unidentified mass peaks appeared after the oxidative modification in Irr (Fig. 6A), suggesting the non-specific oxidation in Irr. Such enhanced non-specific oxidation would be due to the iron release from the heme degradation in Irr. PerR has an intrinsic non-heme iron at the His-cluster region and the oxidation sites were highly localized^28^, while the non-heme iron site of Irr would be formed by the active site conversion, binding the released iron from the degraded heme, and the released iron is likely non-specifically trapped at some amino acid residues near the heme binding site. The multiple non-heme iron sites would result in the less specific oxidative modification in Irr.

Although the formation of 2-oxohistidine in the oxidative modification of Irr indicates that the reactive oxygen species required for the oxidation of amino acids of Irr would be ·OH, the addition of a specific scavenger for ·OH failed to suppress the oxidation of Irr (Fig. 1B). The oxidative modification in PerR was not suppressed by the addition of the specific scavenger for ·OH either^25^. It should be noted here that the oxidative modification site in Irr is ^63^His (Fig. 7A), which corresponds to the oxidized histidine in PerR, ^37^His, near the non-heme iron binding site (Fig. 7A,B), and ^63^His would also be located at the non-heme iron binding site^9^. Histidine residues located near the metal binding site are often the targets for the metal-catalyzed oxidation reactions^23^,^29^. Thus, Irr can activate H_2_O_2_ to highly reactive ROS, such as ·OH, at the non-heme iron binding site in the His-cluster region, and this ROS would immediately attack one of the nearby histidines, ^63^His, before it would be trapped by an ROS scavenger in the solvent, as found for the oxidative modification in PerR^25^.

**Figure 8:**
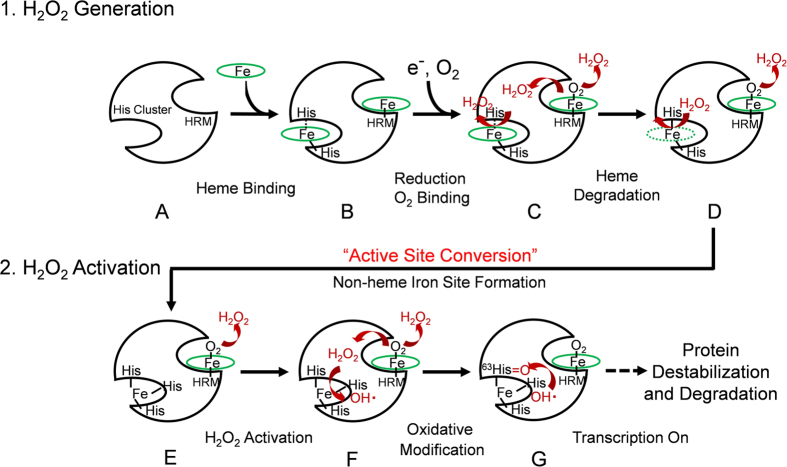
A schematic representation of the mechanism for the oxidative modification of Irr. H_2_O_2_ generation (upper scheme) and H_2_O_2_ activation steps (lower scheme).

Our present study revealed that Irr has two active centers for the self-oxidative modification: heme in the HRM as the primary H_2_O_2_-generation site and non-heme iron in the His-cluster region as the H_2_O_2_-activation site. These findings demonstrate that the heme-responsive transcription factor, Irr, has an unprecedented heme-mediated two-step self-oxidation mechanism involving the active site conversion, as depicted in Fig. 8. In the first step, the H_2_O_2_ generation step, Irr (Fig. 8A) binds heme to form heme-bound Irr (Fig. 8B). In the presence of a reductant, heme is reduced to bind molecular oxygen, which is converted to superoxide ion as a result of autooxidation and then disproportionated to the primary active species, H_2_O_2_ (Fig. 8C). Both of the heme binding sites generate H_2_O_2_, but due to the different heme environments between two heme binding sites, the generation of H_2_O_2_ was more effective in the heme binding site of the HRM region, while the heme in the His cluster region would have higher heme degradation activity by H_2_O_2_. Thus, some of the generated H_2_O_2_ reacts with heme in the His-cluster region, and this reaction causes the release of an iron ion by the degradation of the heme molecule (Fig. 8D). A non-heme iron binding site would then be formed by the active site conversion from heme in the His cluster region (Fig. 8E).

In the second step, the H_2_O_2_ activation step, the H_2_O_2_ that is generated in the HRM region is activated to highly active oxygen species, such as ·OH, at the H_2_O_2_ activation site, where iron released by the heme degradation is liganded to histidines in the His-cluster region (Fig. 8F). The highly reactive species that are generated immediately attack the ligands or adjacent amino acid residues in the His-cluster region, such as ^63^His, resulting in the destabilization of the protein structure and, subsequently, protein degradation (Fig. 8G). The protein degradation of Irr causes the derepression of the target gene, *hemB*, to resume the heme biosynthesis process.

In summary, our work have revealed that Irr is a novel and unique self-oxidative oxidase-transcription factor in which the transcriptional activity is regulated by self-oxidation utilizing an activation of molecular oxygen by heme, demonstrating that heme functions as a signaling molecule for the heme biosynthesis in *Bradyrhizobium japonicum*.

## Experimental Procedures

### Preparation of Irr

Wild-type and mutant Irr proteins were expressed as maltose-binding protein (MBP) fusion proteins using the pMAL fusion protein and purification system (New England BioLabs). The coding region of Irr was amplified by PCR from pCYB1^9^, and the PCR fragment was ligated into pMAL-p2X, which was designed to express fusion proteins with an N-terminal MBP tag. We made Irr mutants, including mutations in the HRM in the N-terminal region (^29^Cys → Ala) and in the His-cluster region in the C-terminal region (^117^His → Ala, ^118^His → Ala, ^119^His → Ala), using a PrimeSTAR mutagenesis basal kit (Takara Bio). The expression and purification of Irr were performed as previously reported^7^. The concentration of Irr was determined using the extinction coefficient, ε = 18.3 mM^−1^cm^−1^ at 280 nm^30^. The heme stock solution was prepared in DMF immediately before use, and the concentration of heme was determined by a pyridine-hemochrome assay^31^ using the extinction coefficient of 191.5 mM^−1^cm^−1^ at 418 nm.

### *In Vitro* Protein Oxidation Analysis

As we previously reported^7^, wild-type Irr can bind two equivalent heme, while only one equivalent of heme is bound for the HRM and His-cluster mutants. The purified Irr, dissolved in 50 mM phosphate buffer (pH 7.4), was incubated with the small excess of two or one equivalent amount of hemin for the wild-type protein and the mutants (the HRM and His-cluster mutants), respectively, dissolved in dimethyl formamide, and the mixture was then passed through a Sephadex G25 column (GE healthcare) to remove free hemin. We prepared 490 μl samples of the heme-bound Irr (20 μM) containing various ROS scavengers: 20 mM mannitol, 500 units/ml Mn-type superoxide dismutase (SOD) from *Bacillus* sp. (Wako Pure Chemical Industries) or 1,000 units/ml catalase from bovine liver (Wako) (all dissolved in 50 mM phosphate buffer). The *in vitro* oxidation reactions of these Irr samples (490 μl) were initiated by 2 mM DTT (dithiothreitol) at room temperature of 20 °C.

The Oxyblot protein oxidation detection kit (Chemicon International) was used to detect the protein carbonyl groups derived from protein oxidation^11^,^32^,^33^. The protein carbonyl groups were derivertized with dinitrophenylhydrazine. The Irr derivatives (20 μl) were loaded onto 15% (w/v) SDS polyacrylamide gels, and electrophoresis was carried out. The immunoblot analyses were performed with a polyclonal rabbit anti-dinitrophenyl antibody (Chemicon) and an HRP-conjugated goat anti-rabbit antibody (Chemicon). The immunoblots were developed using enhanced chemiluminescence substrates (ECL, GE healthcare) followed by visualization with a Fuji LAS3000 CCD camera (Fujifilm). The band intensities of oxidized Irr after the DTT treatment were subtracted from the intensities of the samples at 0 min. The subtracted intensities were expressed relative to the maximum band intensity of the reaction without ROS scavengers (60 or 90 min after the incubation), which was normalized to a value of 1.0.

### Spectroscopic Assay for the Generation of H_2_O_2_ in the Oxidation Reaction of Irr

The spectroscopic assay for the generation of H_2_O_2_ in the oxidation of Irr was conducted as previously reported^14^ using a quantitative peroxide assay kit, Peroxoquant (Pierce). To minimize the concentration of free hemin in the solution, equivalent of heme was added to the purified wild type or mutant Irr solution. 40 μM of heme bound Irr and 40 μM horse myoglobin (Sigma) (typically in 500 μL) were incubated for 10 min with 2 mM DTT. After the incubation, 40 μL aliquots of the samples were added to 360 μL of a mixture of 125 μM xylenol orange and 25 mM FeCl_2_ for 15 min. H_2_O_2_ oxidizes ferrous iron (Fe^2+^) to ferric iron (Fe^3+^) via the Fenton reaction, and the generated Fe^3+^ forms a purple complex with the xylenol orange that exhibits an absorbance around 500–600 nm. We quantified the amount of the generated H_2_O_2_ by the absorbance at 595 nm (ε = 1.5 × 10^4^ M^−1^cm^−1^).

### Protein Oxidation and Mass Spectrometry

For the oxidation reaction, 40 μM of heme-bound wild type or mutant Irr was incubated with 80 μM ferrous ion and 400 μM H_2_O_2_ at room temperature. Before mass spectrometry of the tryptic peptides of Irr, the samples (100 μl) were denatured with 400 μl of the acidic denaturing solution (31.3% methanol, 1.3% acetic acid) containing 62.5 mM EDTA. After passing through a Sephadex G25 column to remove excess ferrous iron, the Irr samples were digested by trypsin at 37 °C for 2 h. The digested samples were mixed with α-cyano-4-hydroxycinnamic acid (Aldrich) as a matrix and analyzed on a Voyager DE-Pro time-of-flight (TOF) instrument (Applied Biosystems). All matrix associated laser desorption ionization time-of-flight mass spectrometry (MALDI-TOF) mass results were obtained in the positive reflector mode. MALDI-TOF mass data were smoothed and calibrated using Data Explorer (Applied Biosystems).

To identify oxidized sites in the Irr peptide, we used an Ultraflex TOF/TOF mass spectrometer equipped with a reflector (Bruker Daltonics GmbH, Bremen). Ions generated using a pulsed UV laser beam (nitrogen laser, 337 nm) were accelerated to a kinetic energy of 23.5 kV. Precursor ions were accelerated to 8 kV and selected in a timed ion gate. Metastable ions generated by the laser-induced decomposition of selected precursor ions were analyzed without any additional collision gas. The fragments accelerated by the 19 kV in the laser-induced forward transfer cell were analyzed after passage through the ion reflector. MALDI-TOF/TOF mass spectra were annotated with the BioTools 2.3 software package (Bruker Daltonics).

### EPR Measurements

The EPR spectra were recorded on a Bruker E-580 spectrometer in the continuous wave mode operating at 9.38 GHz. An Oxford liquid helium flow cryostat was used for cryogenic measurements at 5 K. The measurements were carried out at an incident microwave power of 1.2 milliwatt with a field modulation of 0.5 mT at 100 kHz. The microwave frequency was monitored by a frequency counter (Bruker SuperX-FT bridge), and the magnetic flux density was determined by a teslameter (Bruker ER 036TM). The sample concentration was about 1 mM in 50 mM Tris, 500 mM NaCl, pH 7.5. To quantitatively analyze the EPR signals, MATLAB software (Mathworks, USA) and EasySpin^34^ were utilized and the detailed procedure for the quantification of the EPR signals and simulation of the EPR spectra are described in Supplementary Information.

## Additional Information

**How to cite this article**: Kitatsuji, C. *et al.* Protein oxidation mediated by heme-induced active site conversion specific for heme-regulated transcription factor, iron response regulator. *Sci. Rep.* **6**, 18703; doi: 10.1038/srep18703 (2016).

## Supplementary Material

Supplementary Information

## Figures and Tables

**Table 1:** H_2_O_2_ Generation in wild-type Irr, mutant Irr, myoglobin and BSA in the presence and absence of heme, DTT and catalase.

Proteins	Heme	DTT	Catalase	H_2_O_2_ (μM)^a^
WT Irr	+	+	−	33.4 (±8.6)
WT Irr	+	+	+	3.0 (±2.0)
WT Irr	−	+	−	2.1 (±0.1)
WT Irr	+	−	−	0.8 (±0.3)
C29A Irr	+	+	−	16.5 (±0.3)
H117-119A	+	+	−	31.3 (±0.3)
Myoglobin	−	+	−	66.2 (±1.4)
BSA	+	+	−	77.3 (±0.8)

^a^40 μM proteins (500 μL) were incubated with 40 μM hemin, 2 mM DTT or 4,000 units/ml catalase for 10 min. The H_2_O_2_ that is generated from the reaction mixtures was assessed using the quantitative peroxide assay kit PeroxoquantTM (Pierce) as described in the *Experimental Procedures* section.
