# Supplementary material for: Protein oxidation mediated by heme-induced active site conversion specific for heme-regulated transcription factor, iron response regulator
Source: Sci Rep. 2016 Jan 5;6:18703. doi: 10.1038/srep18703 (PMC4700492; doi:10.1038/srep18703)
Supplement: Supplementary Information [file srep18703-s1.pdf]

Supplementary information

Protein oxidation mediated by heme-induced active site conversion specific for heme-regulated transcription factor, iron response regulator

Chihiro Kitatsuji[1, 2], Kozue Izumi[1], Shusuke Nambu[3], Masaki Kurogochi[4], Takeshi Uchida[1], Shin-Ichiro Nishimura[4], Kazuhiro Iwai[5,6,#], Mark R. O'Brian[7], Masao Ikeda-Saito[3], and Koichiro Ishimori[1,*]

Inductively coupled plasma atomic emission spectrometry (ICP-AES) measurements of Irr. To avoid contamination of trace metals from buffers, the Irr samples were prepared with Chelex 100 (Bio-Rad) treated buffers. The metal content of Irr was determined by inductively coupled plasma atomic emission spectrometry (ICP-AES) using an Optima 2000 DV ICP-AES (Perkin-Elmer, USA) and commercially available standards. The contents of the metals found for typical metal binding proteins, Fe, Zn, Cu, Mn and Ni, are listed in Table S1.

Table S1. Metal Stoichiometry of Purified Irr.

Metal	Number of metals/monomer Irr
Fe	0.14 ± 0.01
Zn	0.013 ± 0.001
Cu	< 0.010
Mn	< 0.0010
Ni	< 0.01

Quantification of EPR spectra. The quantification analysis of the EPR spectra for heme bound Irr before and after the oxidation was done by MATLAB R2015b software (Mathworks, USA). The observed EPR spectra were integrated and the integrated spectra (calculated data points from the observed spectral data are shown in Figure S1) was fitted by 5-component Gaussian functions (solid lines in Figure S1). The parameters for the best fittings were determined by the non-linear least square method and summarized in Table S2. The assignments of the components were conducted by the position of the center field, B ($B < 0.1510$: High Spin Heme, $0.1510 < B < 2000$: Non Heme iron, $2000 < B < 3000$: Low Spin Heme).

The relative contents of the high spin heme, low spin heme, and non-heme iron were estimated by the coefficients of the Gaussian functions, A (Amplitude), in Table S2. Before the oxidation, sum of the coefficients of the high spin species is 7.549 (= 2.648 + 2.562 + 2.339), while the coefficients of the low spin species and unknown species are 3.423, and 1.256, respectively. The relative content of the high spin species is, therefore, 7.549/(7.549 + 3.423 + 1.256) = 0.6174, and that of the low spin heme is 3.423/(7.549 + 3.423 + 1.256) = 0.2799 before the oxidation. After the oxidation, the relative contents of the high and low spin hemes are (2.097 + 0.8119)/(2.097 + 0.8119 + 0.9667 + 1.891 + 1.127) = 0.4220, 1.127/(2.097 + 0.8119 + 0.9667 + 1.891 + 1.127) =0.1635, respectively. The relative content of the non-heme iron after the oxidation is (0.9667 + 1.891)/(2.097 + 0.8119 + 0.9667 + 1.891 + 1.127) = 0.4145.

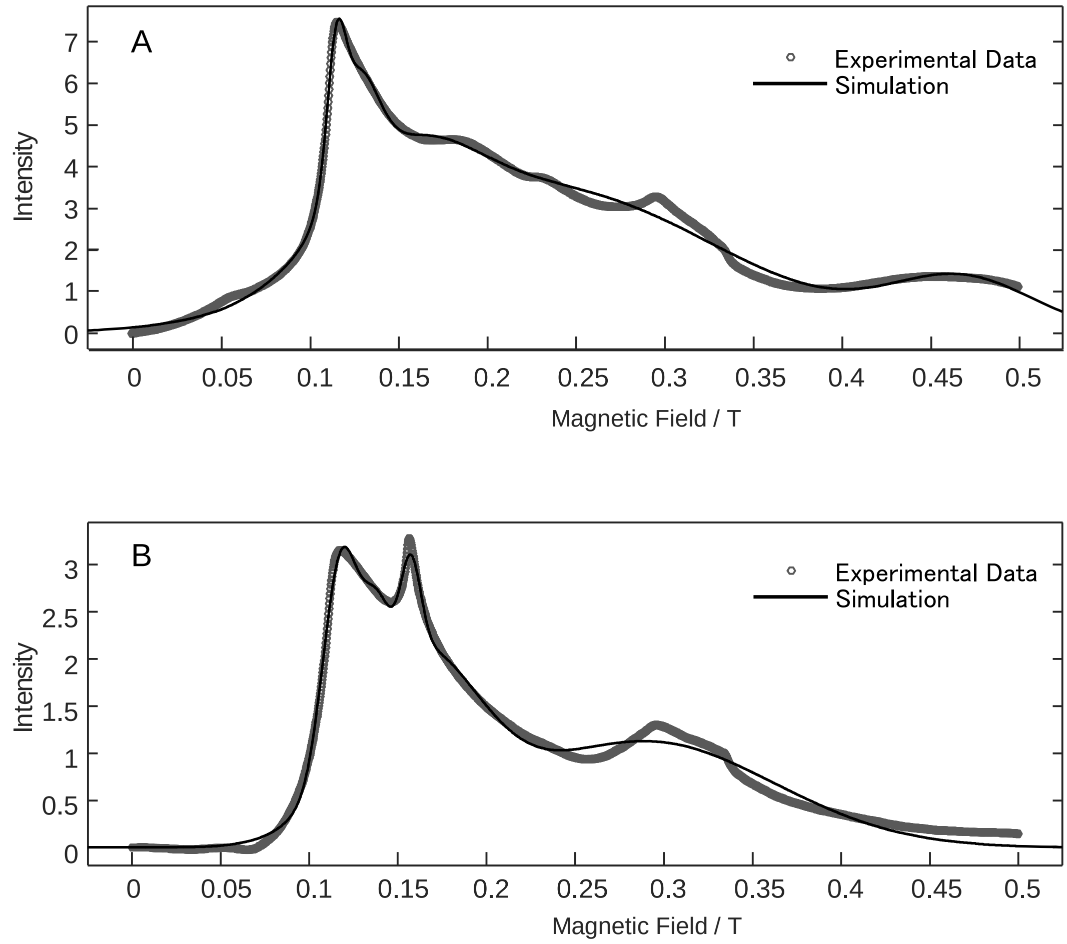

Figure S1 **Integrated EPR spectra for heme-bound Irr.** Before addition of DTT (A), and 6 hours after addition of DTT (B). Integration of the EPR spectra were done by MATLAB R2015b software.

Table S2 Parameters of Simulated EPR Spectrum for Heme-bound Irr Before and After Oxidation

Before Oxidation	Component-1	Component-2	Component-3	Component-4	Component-5
Amplitude (A)[a]	2.648 ± 0.181	2.562 ± 0.079	2.339 ± 0.121	3.423 ± 0.038	1.256 ± 0.022
Center Field (B)[a]	0.1151 ± 0.0002	0.1261 ± 0.0008	0.1504 ± 0.0011	0.2364 ± 0.0016	0.4685 ± 0.0014
Width (C)[a]	$7.024 \times 10^{-3} \pm 3.81\times10^{-4}$	$1.668 \times 10^{-2} \pm 7.1\times10^{-4}$	$5.626 \times 10^{-2} \pm 1.41\times10^{-3}$	$1.329 \times 10^{-1} \pm 1.4\times10^{-3}$	$5.818 \times 10^{-2} \pm 2.37\times10^{-3}$
Assignment	High Spin Heme	High Spin Heme	High Spin Heme	Low Spin Heme	Not Determined

After Oxidation	Component-1	Component-2	Component-3	Component-4	Component-5
Amplitude (A)[a]	2.097 ± 0.082	0.8119 ± 0.827	0.9667 ± 0.0593	1.891 ± 0.037	1.127 ± 0.009
Center Field (B)[a]	0.1174 ± 0.0008	0.1376 ± 0.0013	0.1571 ± 0.0005	0.1593 ± 0.0009	0.2896 ± 0.0016
Width (C)[a]	$1.370 \times 10^{-2} \pm 7.3\times10^{-4}$	$1.127 \times 10^{-2} \pm 1.93\times10^{-3}$	$7.964 \times 10^{-3} \pm 5.74\times10^{-4}$	$5.012 \times 10^{-2} \pm 8.5\times10^{-4}$	$1.024 \times 10^{-2} \pm 2.0\times10^{-4}$
Assignment	High Spin Heme	High Spin Heme	Non Heme Iron	Non Heme Iron	Low Spin Heme

[a]These parameters are defined as follows: $y = A\exp\left\{-\left((x-B)/C\right)^2\right\}$, x : Magnetic Field, y : Intensity of Integrated EPR Signal

A.

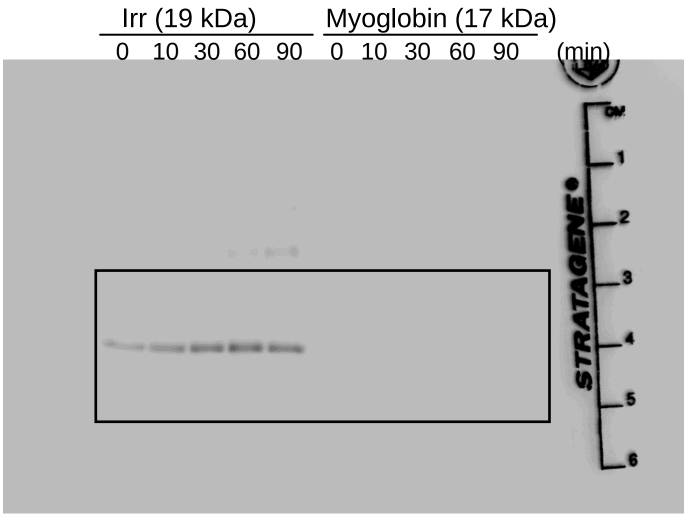
Irr (19 kDa)
Myoglobin (17 kDa)
0 10 30 60 90 0 10 30 60 90 (min)
STRATAGENE

B.

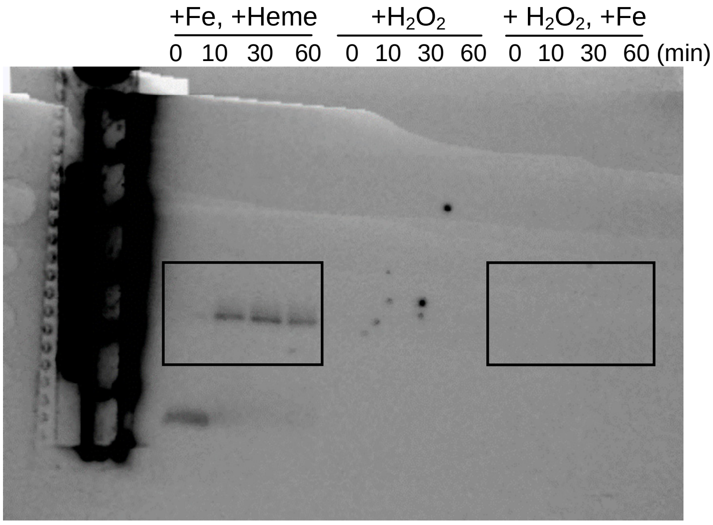
+Fe, +Heme
+H_2O_2
+ H_2O_2, +Fe
0 10 30 60 0 10 30 60 0 10 30 60 (min)

C.

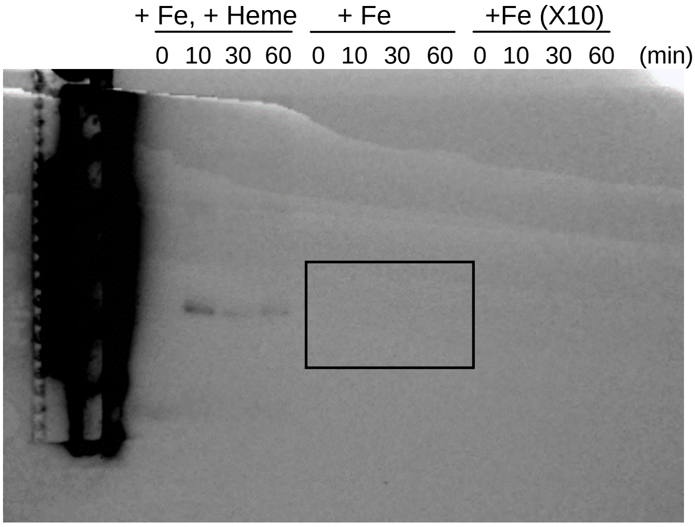
+ Fe, + Heme
+ Fe
+Fe (X10)
0 10 30 60 0 10 30 60 0 10 30 60 (min)

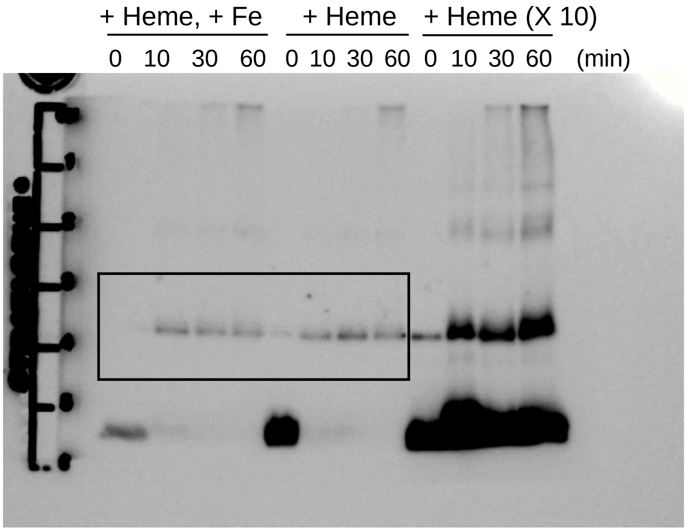

D.

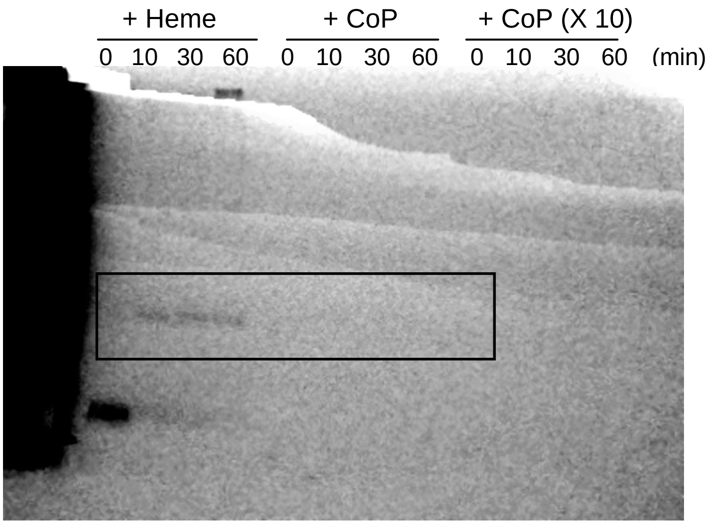

Figure S2. **Full-length blots/gels of Figure 3.** Black frames represent the regions shown in Figure 3. "(X10)" denotes the lane where the applied volume of the sample was 10 times larger than other lanes.
